# Oncostatin M suppresses *IL31RA* expression in dorsal root ganglia and interleukin-31-induced itching

**DOI:** 10.3389/fimmu.2023.1251031

**Published:** 2023-11-16

**Authors:** Masataka Suehiro, Tomofumi Numata, Ryo Saito, Nozomi Yanagida, Chie Ishikawa, Kazue Uchida, Tomoko Kawaguchi, Yuhki Yanase, Yozo Ishiuji, John McGrath, Akio Tanaka

**Affiliations:** ^1^Department of Dermatology, Institute of Biomedical and Health Sciences, Hiroshima University, Hiroshima, Japan; ^2^Department of Pharmacotherapy, Graduate School of Biomedical and Health Sciences, Hiroshima University, Hiroshima, Japan; ^3^Department of Dermatology, The Jikei University School of Medicine, Tokyo, Japan; ^4^St John’s Institute of Dermatology, King’s College London, London, United Kingdom

**Keywords:** oncostatin M, atopic dermatitis, IL-31, itch, pruritus, IL31RA

## Abstract

**Background:**

Atopic dermatitis (AD) is a chronic inflammatory skin disease characterized by intermittent itchy rash. Type 2 inflammatory cytokines such as interleukin (IL)-4, IL-13, and IL-31 are strongly implicated in AD pathogenesis. Stimulation of IL-31 cognate receptors on C-fiber nerve endings is believed to activate neurons in the dorsal root ganglion (DRG), causing itch. The IL-31 receptor is a heterodimer of OSMRβ and IL31RA subunits, and OSMRβ can also bind oncostatin M (OSM), a pro-inflammatory cytokine released by monocytes/macrophages, dendritic cells, and T lymphocytes. Further, OSM expression is enhanced in the skin lesions of AD and psoriasis vulgaris patients.

**Objective:**

The current study aimed to examine the contributions of OSM to AD pathogenesis and symptom expression.

**Methods:**

The expression levels of the OSM gene (*OSM*) and various cytokine receptor genes were measured in human patient skin samples, isolated human monocytes, mouse skin samples, and mouse DRG by RT-qPCR. Itching responses to various pruritogens were measured in mice by counting scratching episodes.

**Results:**

We confirmed overexpression of *OSM* in skin lesions of patients with AD and psoriasis vulgaris. Monocytes isolated from the blood of healthy subjects overexpressed *OSM* upon stimulation with IL-4 or GM-CSF. Systemic administration of OSM suppressed *IL31RA* expression in the mouse DRG and IL-31-stimulated scratching behavior. In contrast, systemic administration of OSM increased the expression of IL-4- and IL-13-related receptors in the DRG.

**Conclusion:**

These results suggest that OSM is an important cytokine in the regulation of skin monocytes, promoting the actions of IL-4 and IL-13 in the DRG and suppressing the action of IL-31. It is speculated that OSM released from monocytes in skin modulates the sensitivity of DRG neurons to type 2 inflammatory cytokines and thereby the severity of AD-associated skin itch.

## 1 Introduction

Eczema or atopic dermatitis (AD) is a chronic inflammatory skin condition manifesting as intermittent flares of itchy skin. The lifetime prevalence is roughly 15%–30% in children and adolescents and 2%–10% in adults (1). The normal barrier function provided by the stratum corneum is deficient in AD, resulting in invasion of allergens or pathogens and leading to activation of lymphocytes, monocytes/macrophages, eosinophils, and dendritic cells. In turn, these immune cells release pro-inflammatory cytokines that stimulate receptors on skin keratinocytes, fibroblasts, and sensory neurons, inducing inflammation and itch (1, 2). In particular, type 2 inflammatory cytokines such as interleukin (IL)-4, IL-13, and IL-31 produced by Th2 cells and type 2 innate lymphoid cells are primary drivers of the inflammatory process (3). As well as causing and prolonging inflammation, these cytokines suppress the production of filaggrin and loricrin by keratinocytes and thereby exacerbate the loss of epidermal barrier function (4). Itching is caused in part by histamine from mast cells, as well as serotonin, interleukins, and thymic stromal lymphopoietin (TSLP) produced by epidermal keratinocytes, which stimulate the C-fiber nerve endings of dorsal root ganglion (DRG) neurons. Interleukin-31 produced by activated CD4+ Th2 cells also activates C-fiber nerve endings of DRG neurons, causing itch (5). In addition to AD, IL-31 is considered the initiating pruritogen involved in itchy skin diseases such as prurigo nodularis, psoriasis vulgaris, and chronic urticaria (6).

The IL-31 receptor is a heterodimer of OSMRβ and IL31RA proteins, and mutations in the OSMR gene encoding OAMRβ cause familial localized cutaneous amyloidosis, a disease associated with intense itch (7, 8), suggesting that OSMRβ is an important signaling component in itch generation. Oncostatin M (OSM), another ligand for OSMRβ, is an inflammatory cytokine released by monocytes/macrophages, dendritic cells, and T lymphocytes under pro-inflammatory conditions (9, 10). The receptor for OSM is a heterodimer of OSMRβ and gp130, and its downstream signals have been found to function in hematopoiesis, mesenchymal stem cell differentiation, liver regeneration, cardiac remodeling, nociception, inflammation, and metabolism (11). However, OSM functions in skin remain unclear. Both OSMRβ and gp130 are expressed in keratinocytes, fibroblasts, and DRG neurons, suggesting that OSM may modulate skin inflammation and sensation by acting on skin component cells and sensory nerves. In fact, expression of OSM is enhanced in AD and psoriasis vulgaris lesions, although the mechanisms underlying this upregulation are unknown. Recently, it was reported that OSM does not directly cause itch but rather induces hypersensitivity to itch by enhancing the neuronal response to the pruritogens histamine and leukotriene (12). In this study, the conditions leading to OSM release by skin monocytes and the effects of OSM on IL-4, IL-13, and IL-31 receptors in skin cells and DRG neurons were examined to elucidate potential contributions to AD pathogenesis and skin pruritus symptoms.

## 2 Methods

### 2.1 Harvesting of skin samples from AD and psoriasis vulgaris patients

Fixed paraffin-embedded biopsy samples of skin from AD and psoriasis vulgaris patients were obtained from Hiroshima University Hospital. All samples (10 from AD patients and 10 from psoriasis vulgaris patients) were extracted within the past three years. Fifteen normal skin tissues from mapping skin biopsies were used as controls. Briefly, samples were extracted using a 4-mm punch biopsy, promptly fixed in formalin, embedded in paraffin, and sectioned at 10 μm thickness. Total RNA was isolated using the RNeasy FFPE kit (Qiagen, Hilden, Germany) for subsequent gene expression analysis by RT-qPCR (Section 2.9).

### 2.2 Purification and culture of human monocytes

Peripheral blood mononuclear cells (PBMCs) were isolated from healthy volunteers in Leucosep™ tubes (Greiner Bio-One Co, Tokyo. Japan) by density gradient centrifugation with Ficoll (GE Healthcare Japan, Tokyo. Japan). Monocytes were then purified from PBMCs using the Human Monocyte Enrichment Kit (STEMCELL Technologies, Tokyo, Japan). Monocytes were cultured in RPMI medium supplemented with 5% human serum at 37°C under a 5% CO_2_ atmosphere for 24 h, then seeded on 24-well plates and treated for 1 h with RPMI medium containing 50 ng/mL of various test substances, including IL-4 (R&D Systems, Minneapolis, MN, USA), IL-13 (R&D Systems), IL-17A (PeproTech, Cranbury, NJ, USA), IL-33 (R&D Systems), IL-10 (PeproTech), GM-CSF (R&D Systems), and TNF-α (R&D Systems).

### 2.3 Mice

All animal experiments were approved by the Institutional Animal Care and Use Committee of Hiroshima University and were performed in accordance with the Guidelines for the Care and Use of Laboratory Animals issued by Hiroshima University. Eight-week-old male C57BL/6 mice were purchased from Charles River Laboratories Japan.

### 2.4 Maintenance of a human epidermal keratinocyte cell line

The keratinocyte cell line Human Epidermal Keratinocytes, pooled (HEK) was obtained from Gibco (Gibco ThermoFisher, NY, USA) and cultured in Keratinocyte SFM (Gibco ThermoFisher) supplemented with 5 μg/mL human recombinant epidermal growth factor (Gibco ThermoFisher), 50 μg/mL bovine pituitary extract (Gibco ThermoFisher), and 100 IU/mL penicillin/streptomycin (Gibco ThermoFisher). Cells were stimulated with recombinant human OSM (100 ng/mL, R&D Systems) for 1, 3, 6, and 24 h, and gene expression changes analyzed by RT-qPCR (Section 2.9). In addition, cells were stimulated with various concentrations of recombinant human OSM (0.1–100 ng/mL, R&D Systems) for 3 h. and gene expression changes were analyzed by RT-qPCR (Section 2.9).

### 2.5 Quantitation of *Osmr* and *Il31ra* expression in isolated (ex vivo) OMS-stimulated mouse DRG

Male C57BL/6 mice aged 8 to 12 weeks were euthanized with carbon dioxide and DRGs promptly dissected out and cultured in Dulbecco’s modified Eagle’s medium (Gibco ThermoFisher) supplemented with 10% fetal bovine serum (Gibco ThermoFisher) and penicillin/streptomycin at 37°C under a humidified atmosphere of 5% CO_2_. Cultured DRGs were stimulated with recombinant mouse OSM protein (100 ng/mL, R&D Systems) for 3, 6, 12, and 24 h, and total RNA was extracted using a RNeasy Fibrous Tissue Mini Kit (Qiagen, Hilden, Germany) for subsequent measurement of gene expression changes.

### 2.6 Quantitation of *Osmr* and *Il31ra* expression in dispersed culture mouse DRGs

DRGs were extracted using the same procedure as in Section 2.5 and sequentially dissociated in collagenase type III (Worthington, California, USA) and trypsin (Gibco ThermoFisher), which digest all intercellular connections. Neurons were sorted using Percoll (GE Healthcare) and cultured for 3 days to adjust cell numbers. Cultured DRGs were stimulated with recombinant mouse OSM protein (100 ng/mL, R&D Systems) for 3, 6, and 24 h, and total RNA was extracted using a RNeasy Fibrous Tissue Mini Kit (Qiagen, Hilden, Germany).

### 2.7 Quantitation of cytokine receptor expression in OMS-stimulated mouse skin and DRGs

Mice were administered four doses of recombinant mouse OSM protein (R&D Systems) at a dose of 200 ng in 50 μL saline every 8 h or equal-volume saline (control) by tail vein injection. Four h after the final injection, all mice were euthanized with carbon dioxide. Skin tissues were extracted from four treated and four control mice using a 4-mm Derma punch^®^ (Maruho, Tokyo, Japan), while DRGs were collected following a previous described method of “Dispersed Cultures of Mature Rat DRG Neurons and Their Applications.”.

### 2.8 ELISA for OSM production

The ELISA assay for human OSM was performed using the Human Oncostatin M (OSM) ELISA kit (Ray Biotech, Peachtree Corners, GA, USA) according to the manufacturer’s protocol.

### 2.9 Isolation of RNA

Total RNA was isolated from paraffin-embedded tissue sections using the RNeasy FFPE kit, from monocytes using the RNeasy Mini Kit, and from DRGs and skin samples was using the RNeasy Fibrous Tissue Mini Kit (all from Qiagen) according to the manufacturer’s instructions.

### 2.10 Real-time quantitative PCR (RT-qPCR)

First-strand cDNA was synthesized from isolated RNA using a QuantiTect^®^ Reverse Transcription Kit (Qiagen), and RT-qPCR was performed using a QuantStudio 3 real-time PCR system (ThermoFisher) with the following thermocycle: denaturation at 95°C for 15 s, annealing at 60°C for 60 s. Expression of the glyceraldehyde 3-phosphate dehydrogenase gene (*GAPDH*) was measured as the internal control. Primer pairs for detecting *OSM,IL31, IL4, IL31RA, OSMR*, *CCL2*, *Il31ra, Osmr, Il4ra, Il13ra1, Il2rg*, and *Il13ra2* were obtained from ThermoFisher Scientific.

### 2.11 Measurement of scratching behavior

Itch-related scratching behavior in treated mice was analyzed using a SCLABA^®^-Real system (Noveltec, Kobe, Japan). Briefly, mice were preinjected with OSM (100 ng) in 50 μL saline via the tail vein. After 11.5 h, four mice were placed in an acrylic cage (19.5 × 24 × 35 cm) for at least 30 min to acclimate then injected with histamine (55.6 μg), serotonin (2.5 μg), or IL-31 (500 ng) in 50 μL saline. Scratching behavior was monitored remotely for 30 or 120 min at an ambient room temperature of 22°C.

### 2.12 Statistical analysis

Data are expressed as the mean ± SD or mean ± SEM as indicated. All statistical analyses were performed using GraphPad Prism 8 (GraphPad Software, Inc., La Jolla, CA, USA). Two sample groups were compared by independent samples t-test and three or more groups by analysis of variance with Tukey’s multiple comparisons tests. A p < 0.05 was considered statistically significant for all tests. In figures, significance level is represented by *p < 0.05, **p < 0.01, ***p < 0.001, and ****p < 0.0001.

### 2.13 Study approval

Mouse experiments were approved by the animal experimentation ethics committee of Hiroshima University and informed-consent was obtained from all human subjects following approval by the ethics committee of Hiroshima University.

## 3 Results

### 3.1 Expression of *OSM* is upregulated in human AD and psoriasis vulgaris lesions

**Figure 1:**
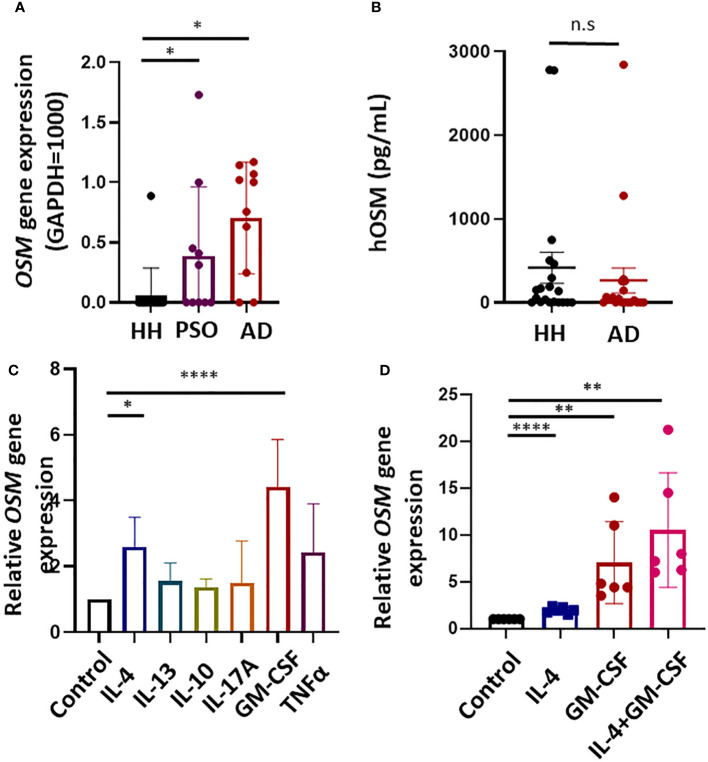
Expression of the gene encoding oncostatin M (*OSM*) is upregulated in atopic dermatitis (AD) lesions and cytokine-stimulated human monocytes. **(A)** Expression of *OSM* was undetectable in 14 of 15 healthy human (HH) skin samples but was detected in 8 of 10 AD lesion samples and 5 of 10 psoriasis (PSO) lesion samples by RT-qPCR. **(B)** OSM protein levels in serum did not differ significantly between healthy humans and patients with AD (n = 20). **(C)** Stimulation of monocytes isolated from healthy humans with IL-4 (50 ng/mL) or GM-CSF (50 ng/mL) increased *OSM* expression (n = 6 samples from 6 human volunteers). **(D)** Stimulation of monocytes from healthy humans with both IL-4 and GM-CSF (50 ng/mL) increased *OSM* (n = 6 monocyte samples from 6 human volunteers). All results presented as mean ± SD. * p < 0.05, **< 0.01 and **** p < 0.0001 by Tukey’s multiple comparisons test.

To elucidate the contributions of OSM signaling to AD and psoriasis vulgaris, we first examined if *OSM* expression is upregulated in lesions compared to healthy skin tissue. Indeed, expression was undetectable in 14 of 15 healthy control skin biopsy samples but detected in 8 of 10 AD lesion samples and 5 of 10 psoriasis vulgaris lesion samples (**Figure 1A**). The expression of *OSMR* in lesional skin showed a tendency to be lower compared to healthy skins, but no significant difference was observed (**Figure S1**). The expression of *IL31RA* could not be detected in human skin tissues (Data not shown).

### 3.2 OSM protein levels in the serum of healthy humans and AD patients

No significant difference in OSM levels was observed among serum samples obtained from 20 patients with AD currently undergoing treatment at our hospital and 20 healthy humans (**Figure 1B**).

### 3.3 Expression of *OSM* is upregulated in healthy human monocytes by IL-4 and CM-CSF stimulation

To investigate if the upregulation of OSM in skin lesions of patients with AD and psoriasis vulgaris results from a local pro-inflammatory cytokine environment, *OSM* expression was measured in human monocytes isolated from healthy human subjects following stimulation by cytokines known to be upregulated in AD and psoriasis lesions. Stimulation with IL-4 or GM-CSF (both at 50 ng/mL for 1 h) significantly increased *OSM* expression compared to vehicle-treated control samples (**Figure 1C**), and co-stimulation with IL-4 and GM-CSF enhanced *OSM* expression (**Figure 1D**).

### 3.4 OSM stimulation modulates IL-31 receptor gene expression in isolated mouse DRG

**Figure 2:**
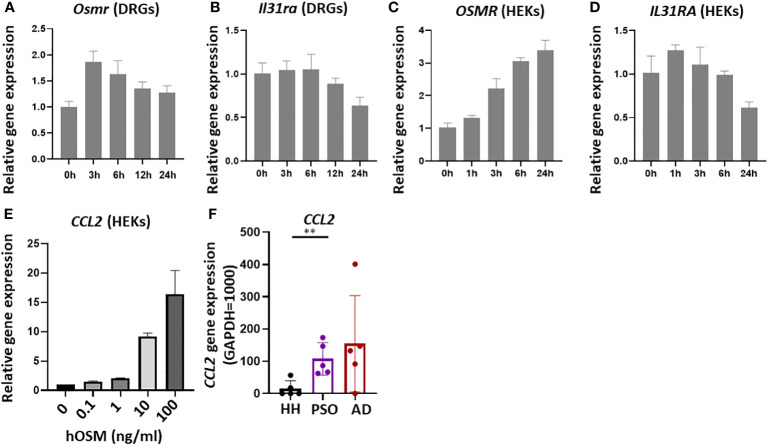
Stimulation of isolated mouse dorsal root ganglia (DRG) and cultured human keratinocytes (HEKs) with OSM modulated IL-31 receptor subunit gene expression. **(A, B)** In isolated mouse DRG, OSM treatment (100 ng/mL) rapidly upregulated *Osmr* expression, followed by a slow return to baseline, and more slowly downregulated *Il31ra* expression. **(C, D)** In HEKs as well, OSM progressively increased *OSMR* expression **(C)** and more slowly reduced *IL31RA* expression **(D)**. **(E)** In HEKs, *CCL2* gene expression increases in an OSM concentration-dependent manner. **(F)** In lesions of AD and psoriasis vulgaris patients, there was a tendency for higher *CCL2* gene expression compared with healthy humans (HH). All results presented as mean ± SD. ** p < 0.01 by unpaired t-test. All experiments except F were performed ≥three times.

**Figure 3:**
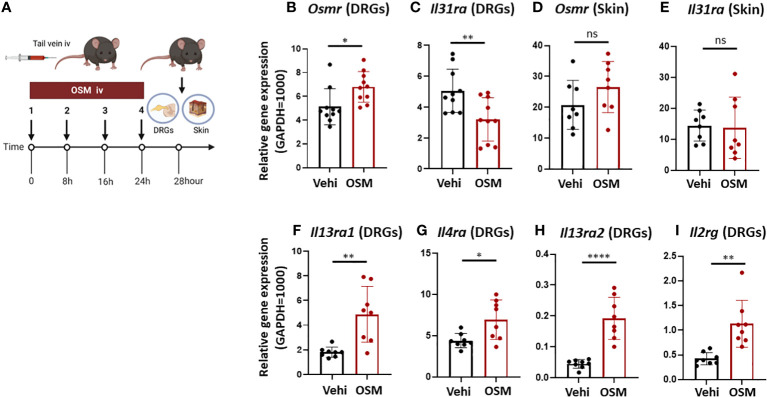
High systemic OSM modulates IL-31 receptor subunit gene expression in mouse DRG. **(A)** Experimental schema. Mice were injected with OSM (200 ng) into the tail vein every 8 h for a total of four doses. At 28 h after the final dose, samples of the dorsal root ganglion (DRG) and dorsal skin were collected. **(B, C)** Injection of OSM increased DRG expression of *Osmr* **(B)** and decreased DRG expression of *Il31ra* **(C)** compared to vehicle (Vehi) injection (n = 10 DRG samples from 10 injected mice). **(D, E)** Alternatively, systemic injection of OSM did not alter IL-31 receptor subunit expression in mouse skin (n = 8 skin samples from 8 mice). **(F–I)** Systemic injection of OSM also increased DRG expression levels of IL-4 and IL-13 receptor subunits (n = 8 DRG samples from 8 mice). All results are presented as mean ± SD. *p < 0.05, ** p < 0.01, and **** p < 0.0001 by unpaired t-test. ns, non-significant.

Stimulation of IL-31 receptors expressed on sensory neurons is believed to promote itch in AD. To investigate the potential contribution of *OSM* upregulation to this process, we examined changes in IL-31 receptor gene expression in isolated mouse skin samples and DRGs treated with OSM (100 ng/mL for up to 24 h). In DRGs, expression of *Osmr* increased immediately after OSM stimulation (**Figure 2A**), while the expression of *Il31ra* decreased starting after 12 h of treatment (**Figure 2B**). In contrast, no expression changes were observed in mouse skin samples (**Figures 3D, E**). Conversely, in dispersed cultured DRGs, there was an observed trend of increased expression for both *Osmr* and *Il31ra* after OSM stimulation (**Figures S2A, B**).

### 3.5 OSM stimulation modulates IL-31 receptor gene expression in cultured HEK

Stimulation of HEKs with OSM upregulated *OSMR* expression and downregulated *IL31RA* expression (**Figures 2C, D**).

### 3.6 OSM stimulation increases *CCL2* gene expression in HEKs

*CCL2* gene expression increased in a concentration-dependent manner upon OSM stimulation (**Figure 2E**). In fact, a notable trend of increased *CCL2* expression was observed in both AD and psoriasis lesions (**Figure 2F**).

### 3.7 OSM stimulation modulates IL-31, IL-4 and IL-13 receptor expression in mouse skin and DRG

To examine if OSM stimulation also modulates the expression of IL-31 receptors and other cytokine receptors *in vivo*, we measured *Osmr* and *Il31ra* expression in mouse skin and DRG samples freshly excised 28 h following the last of 4 OSM systemic injections (200 ng at 8-hour intervals in the tail vein) (**Figure 3A**). Consistent with observations in culture, the expression of *Osmr* was upregulated (**Figure 3B**) while that of *Il31ra* was downregulated in freshly excised DRG tissue (**Figure 3C**). In contrast, there were no significant changes in *Osmr* and *Il31ra* expression in skin tissue (**Figures 3D, E**), suggest that longer-term changes in IL-13 signaling occur at the level of the neuronal cell body. Furthermore, OSM also increased the expression of genes encoding IL-4 and IL-13 receptor subunits IL-4Rα, IL-4Rγc, IL-13Rα1, and IL-13Rα2 (*Il4ra*, *Il2rg*, *Il13ra1*, *Il13ra2*) in freshly excised mouse DRG (**Figures 3F–I**).

### 3.8 OSM suppresses scratching behavior in mice

**Figure 4:**
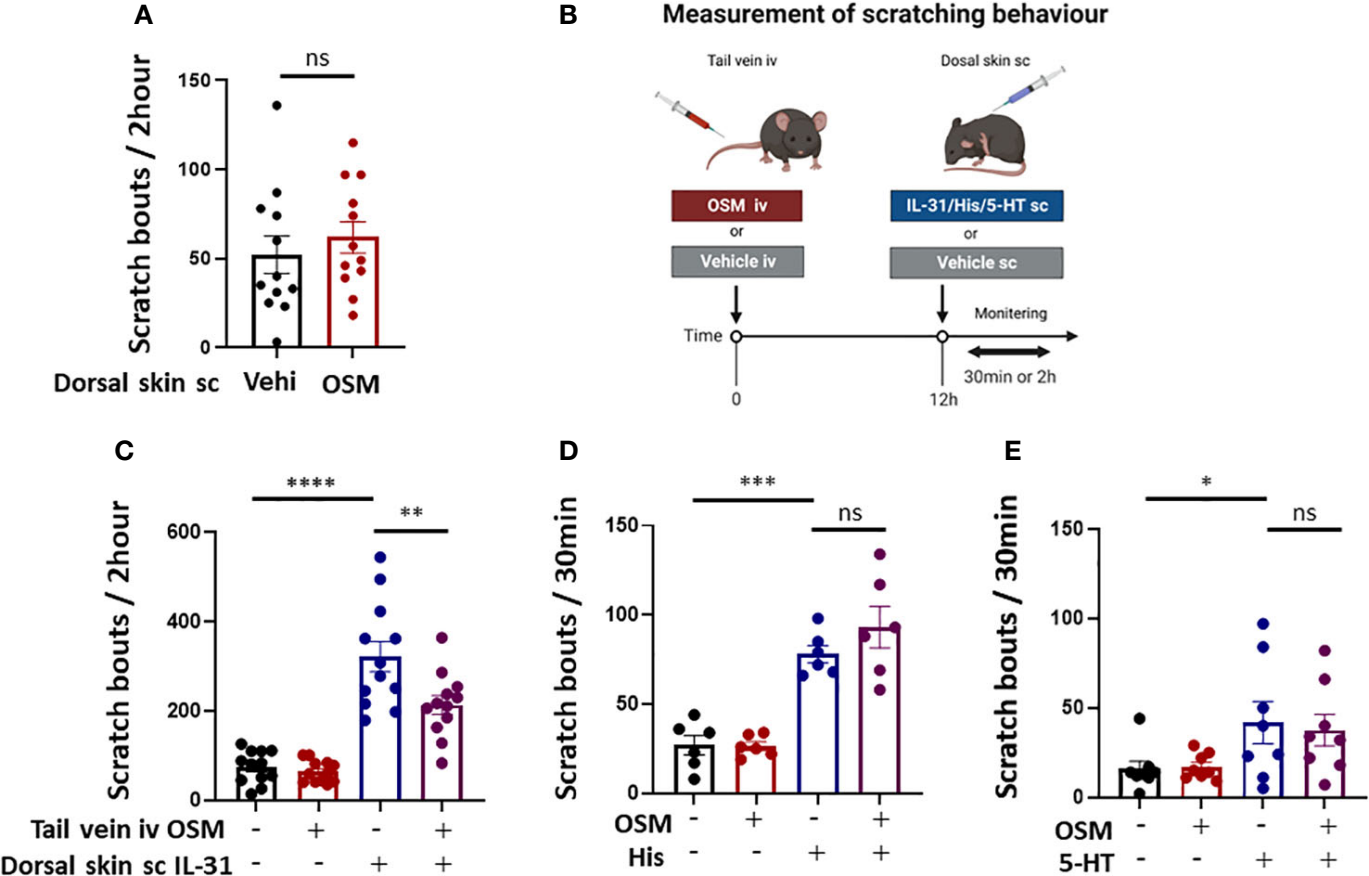
Systemic OSM injection suppresses IL-31-induced scratching behavior in mice. **(A)** Intradermal administration of OSM alone did not induce itching. (n = 12 mice per treatment group). **(B)** The schedule of OSM and pruritogen administration followed by monitoring of scratching behavior. **(C)** Intravenous (iv) injection of OSM into the tail vein 12 h prior to subcutaneous (sc) injection of IL-31 reduced scratching behavior compared to iv vehicle (Vehi) injection (n = 12 mice per treatment group). **(D, E)** Tail vein injection of OSM did not alter scratching behavior induced by sc injection of **(D)** histamine (His) or **(E)** serotonin (5-HT) (n = 6 mice per treatment group). All results expressed as mean ± SEM. * p < 0.05, ** p < 0.01, *** p < 0.001, and **** p < 0.0001 by Tukey’s multiple comparisons test. n.s, non-significant.

Based on our findings that OSM reduced IL-31RA expression in mouse DRG, we predicted that the behavioral response to IL-31 would be reduced by OSM stimulation. For these tests, 100 ng of OSM was injected into the tail vein 8 h prior to subcutaneous treatment with various pruritogens including IL-31. To first examine if OSM alone initiates itch, scratching behavior was compared between mice receiving OSM or saline starting 2-h post treatment. The number of scratch events was slightly but not significantly higher following OSM administration compared to saline administration (61.9 ± 8.8 vs. 52.1 ± 10.5) (**Figure 4A**), suggesting little direct pruritogenic action. However, systemic pretreatment with OSM did suppress subsequent IL-31-induced itch (**Figure 4B**) as the number of scratching events observed over a 2-h period following IL-31 administration was significantly lower among mice receiving OSM (100 ng) 12 h earlier compared to mice receiving saline (213.8 ± 21.0 times vs. 321.7 ± 34.0 times) (**Figure 4C**). Alternatively, systemic OSM had no effect on scratching behavior induced by histamine or 5-HT (**Figures 4D, E**).

## 4 Discussion

This study provides evidence that oncostatin M can reduce cytokine-induced itch associated with AD by suppressing pro-inflammatory cytokine signaling in keratinocytes and sensory neurons. This is the first report demonstrating the negative regulatory role of OSM in itching. OSM may be an important endogenous negative regulator of AD pathogenesis and symptom expression.

The expression of *OSM* was substantially elevated in skin lesions from AD and psoriasis vulgaris patients (**Figure 1**), consistent with a previous report (13). Multiple inflammatory cell types infiltrate AD and psoriasis vulgaris lesions, including OSM-producing monocytes/macrophages. Monocytes are known to release OSM in response to GM-CSF stimulation (14). In the present study as well, GM-CSF and IL-4 enhanced *OSM* expression in isolated human monocytes, and there is a trend toward higher CCL2 expression in the skin lesions of AD and psoriasis vulgaris (**Figure 2**). In the acute phase of AD, there is a substantial increase in the number of cells in skin expressing IL-4, IL-5, and IL-13 mRNAs, while in the chronic phase, skin lesions exhibit greater numbers of cells expressing IL-5, GM-CSF, IL-12, and IFN-α mRNAs (15). Monocytes differentiate into dendritic cells in the presence of GM-CSF and IL-4 (16–18), suggesting that the chronic phase of AD is associated with the differentiation of OSM-expressing monocytes into dendritic cells. Furthermore, keratinocytes produce CCL2 upon OSM stimulation, which attracts additional monocytes into the dermis (19). We suggest that monocytes and keratinocytes are mutually activated via OSM and CCL2 release in the presence of IL-4 and GM-CSF within AD skin lesions.

It has been reported that IL-31 transmits itch stimuli to the central nervous system by binding receptors expressed on DRG neurons and activating the ERK signaling pathway (20). Consistent with this mechanism, systemic OSM treatment reduced IL-31-induced scratching behavior in mice. Interleukin-31 is believed to act as a pruritogenic agent in multiple skin diseases. In fact, the humanized anti-IL31RA nemolizumab was recently approved in Japan for the relief of AD-associated pruritus. In contrast to IL31-induced itch, OSM had no effect on itch induced by histamine or serotonin. Endogenous itch caused by histamine, leukotrienes, and serotonin is also dependent on stimulation of cognate receptors expressed by keratinocytes and DRGs (21, 22). Thus, the effects of OMS appear specific to IL-31 receptor expression and itch transmission.

It is speculated that keratinocytes and DRG neurons expressing IL-31 receptors directly contribute to itch and that the associated transduction pathways are modulated by various other factors, including other cytokines, through effects on IL-13 receptor activity. Miake and colleagues reported that IL-4 stimulation enhanced *IL31RA* gene expression and IL31-induced production of CCL17 and CCL22 in BMDCs (23), suggesting that *IL31RA* expression level influences the responsiveness of cells to IL-31. Tail vein administration of OSM enhanced the expression of *Osmr* but suppressed *Il31ra* expression in the mouse DRG (**Figures 3B, C**). In mouse skin tissue, however, the expression levels of these receptor-related genes were unchanged by OSM, whereas OSM stimulation enhanced the expression of *OSMR* and suppressed *IL31RA* expression in primary human cultured keratinocytes (HEKs) (**Figures 2C, D**, **3D, E**). Furthermore, systemic OSM suppressed phosphorylation of ERK by IL-31 in HEKs. We speculate that OSM may suppress IL-31-induced pruritus by reducing IL31RA expression and downstream ERK activity in both DRGs and keratinocytes. In contrast, OSM stimulation enhanced the expression of receptor subunits for IL-4 and IL-13 in the mouse DRG (**Figures 3F–I**). Both IL-4 and IL-13 receptors are also upregulated in the lesional skin of AD patients (24), suggesting that OSM suppress itch by promoting IL-4 and -13 responses and by concomitantly suppressing IL-31 responses in the DRG.

Previous reports have concluded that OSM acts as a modulator rather than an initiator of itch. Tseng and colleagues simultaneously administered pruritogenic substances and OSM subcutaneously to mice and found that OSM enhanced histamine- and leukotriene C4 (LTC4)-induced itch (12). In mouse DRG, *Il31ra* gene expression was not immediately affected by OSM stimulation but decreased substantially after 12 h (**Figure 2B**). Alternatively, expression levels of histamine H1 and H4 receptor genes were not influenced by OSM (data not shown). Systemic administration of OSM 12 h before subcutaneous administration of pruritogenic substances (i.e., a time sufficient to downregulate *Il31ra*) suppressed IL-31-induced scratching behavior, but did not alter histamine- or serotonin-induced scratching behavior. The discrepancies between the present study and previous reports may be explained by the different intervals between systemic OSM and subcutaneous pruritogen administration, as OSM-induced modulation of histamine receptor subunit expression is rapid but transient and so may have subsided after 12 h. The effects of OSM on pruritic stimulation pathways are likely to be diverse and require further validation.

Primary sensory neurons in the DRG have been classified into 11 subsets based on comprehensive transcriptome analysis, with only one type expressing *IL31RA* and two mechanoreceptive subtypes expressing OSM receptors (25). In DRGs, 50.6% of small-diameter neurons (<30 µm) expressed IL31RA, whereas expression was not detected in the more numerous large-diameter neurons (>50 µm). As a result, only a small proportion of DRG neurons (< 4%) responded to IL-31 (20). In the current study, OSM stimulation of isolated mouse DRGs reduced *IL31RA* expression (**Figures 2C, D**), whereas in primary cultured DRG neurons, *IL31RA* expression was increased by OSM (**Figure S2**). During culture, the number of differentiated DRG neurons decreased and undifferentiated DRG neurons with proliferative potential increased and eventually were the majority. Therefore, cultures may not be a reliable model to assess cytokine responses in DRG neurons. Future work is needed to examine whether there are differences in cellular responses to OSM stimulation among DRG neuron subtypes.

This study has several limitations. It is not known whether OSM can suppress IL-31-induced itch in humans for AD treatment. The correlation between the degree of itching in atopic dermatitis and psoriasis vulgaris and the level of OSM in the skin or serum remains unclear and is a subject for further investigation. We also need to confirm that IL31RA surface expression (like mRNA expression) is reduced by OSM and elucidate the specific signaling pathways involved. Finally, the safety of OSM treatment and potential effects on other sensory processes requires further investigation.

**Figure 5:**
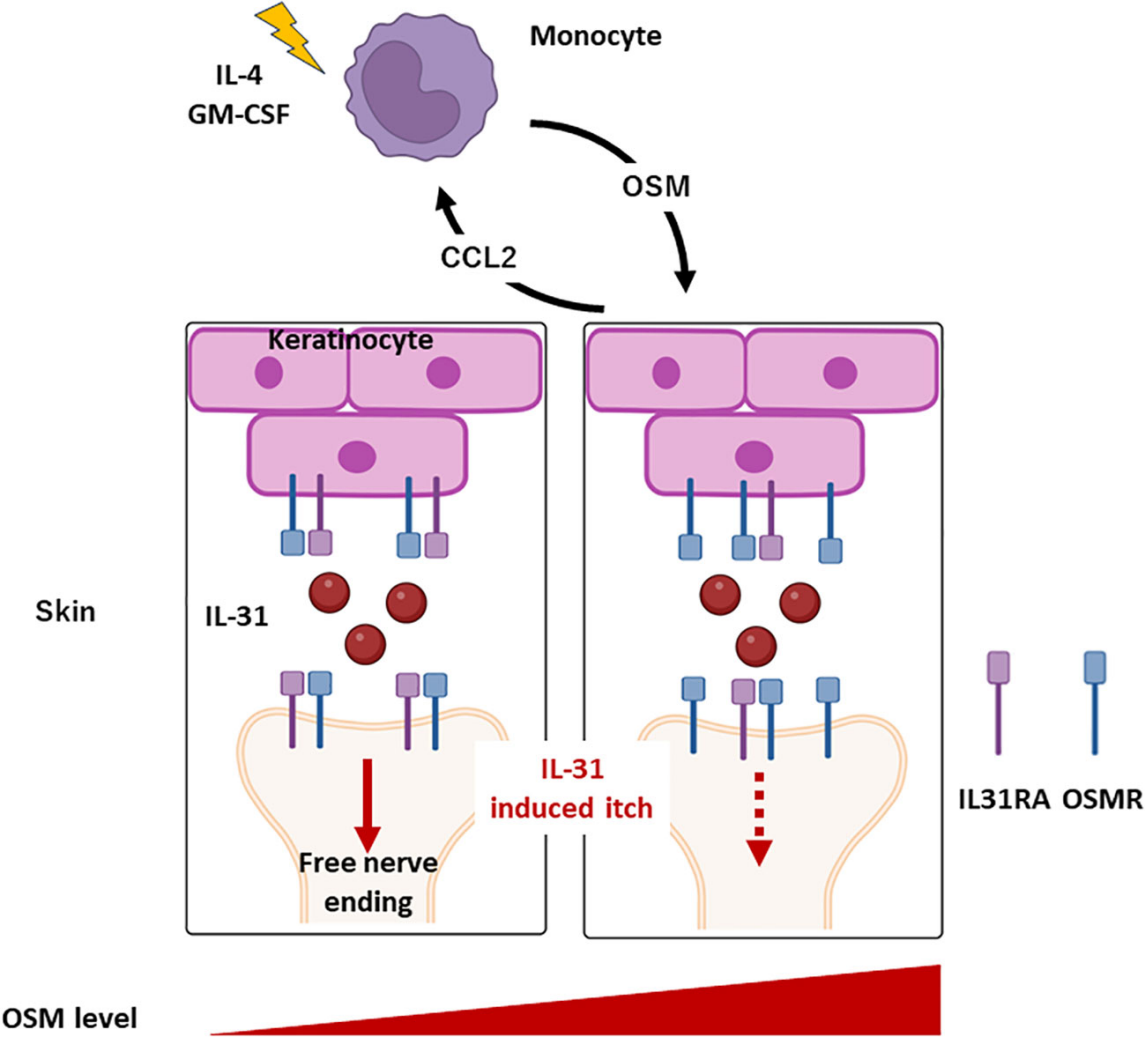
Roles of monocyte and OSM in AD. Monocytes produce OSM in response to stimulation by IL-4 and GM-CSF. OSM triggers the release of CCL2 from keratinocytes, inducing monocytes infiltration into the skin. Under conditions of elevated OSM in skin, *IL31RA* expression is downregulated while *OSMR* expression is upregulated in keratinocytes and DRG. Consequently, suppression of IL-31-induced itch is triggered.

In the present study, we confirmed that *OSM* expression is upregulated in AD lesions and further demonstrated that upregulation can be induced in skin cells by IL-4 and GM-CSF. Systemic OSM elevation was found to reduce IL-31 receptor subunit expression in the mouse DRG as well as IL-31-induced scratching behavior (**Figure 5**). At the same time, OSM enhanced the expression of IL-4 and IL-13 receptor subunits in the DRG. Collectively, these findings suggest that OSM contributes to AD pathogenesis and symptom expression by modulating the expression of IL-31, IL-4, and IL-13 receptor subunits on DRG neurons, thereby altering intracellular signaling pathways responsible for regulating the transmission of itch signals to the central nervous system.

## Data availability statement

The datasets presented in this study can be found in online repositories. The names of the repository/repositories and accession number(s) can be found in the article/**Supplementary Material**.

## Ethics statement

The studies involving humans were approved by Hiroshima University Clinical Research Review Committee. The studies were conducted in accordance with the local legislation and institutional requirements. The participants provided their written informed consent to participate in this study. The animal study was approved by Hiroshima University Animal Experimentation Committee. The study was conducted in accordance with the local legislation and institutional requirements.

## Author contributions

MS and AT made contributions to the conception of the work and were major contributors in writing the manuscript. MS, TN, RS, KU, TK, and AT collected the data. YY, YI, and JM supervised the project and confirmed the authenticity of all raw data. All authors contributed to the article and approved the submitted version.

## Supplementary material

Supplementary Figure 1 Gene expression in lesions of AD and psoriasis vulgaris patients. *OSMR* gene expression tends to be high in healthy humans (n = 5). Although *IL31*, *IL4*, *GM-CSF* and *IL31RA* gene expression was investigated, expression could not be detected.

Supplementary Figure 2 Oncostatin M modulated the expression levels of *Osmr* and *Il31ra* in dispersed cultured mouse DRGs. **(A)** The expression of *Osmr* increased with time after OSM stimulation. **(B)** The expression of *Il31ra* increased up to 3 h and then gradually decreased below baseline. All experiments were performed ≥three times.
