# Supplementary figures and images for: Oncostatin M suppresses IL31RA expression in dorsal root ganglia and interleukin-31-induced itching
Source: Front Immunol. 2023 Nov 16;14:1251031. doi: 10.3389/fimmu.2023.1251031 (PMC10687395; doi:10.3389/fimmu.2023.1251031)

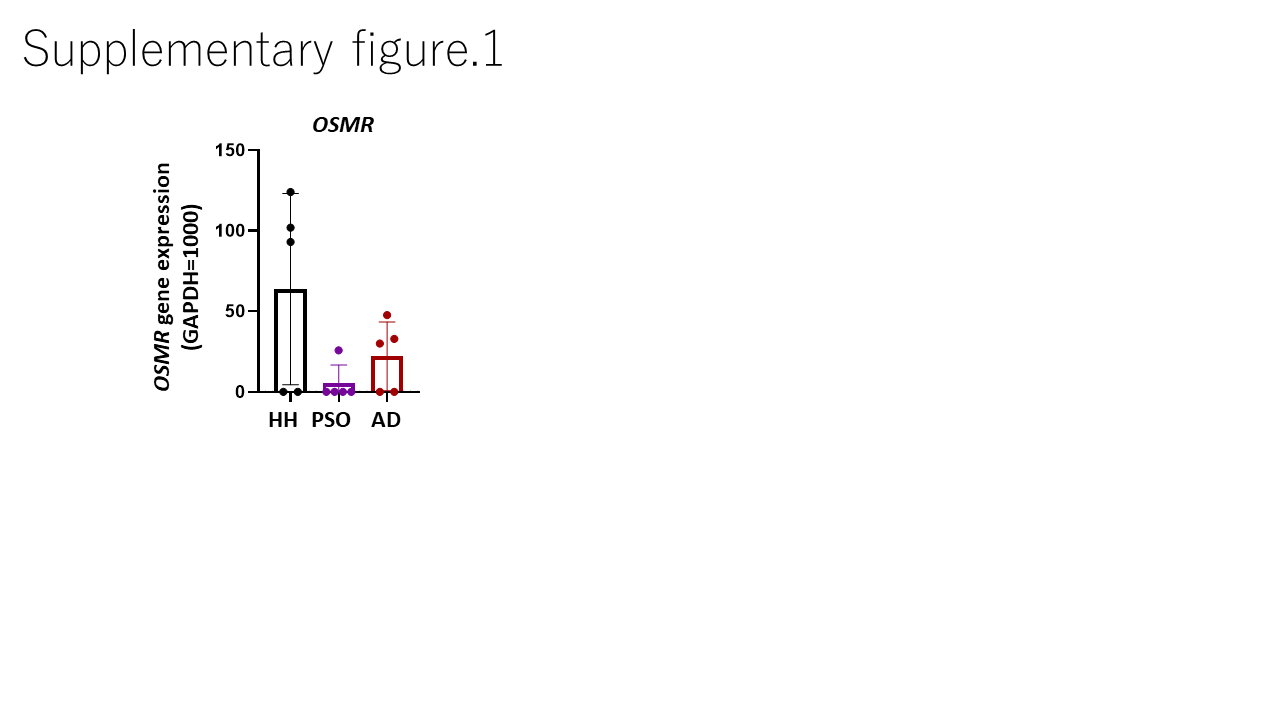

Supplement: Supplementary Figure 1 — Gene expression in lesions of AD and psoriasis vulgaris patients. OSMR gene expression tends to be high in healthy humans (n = 5). Although IL31, IL4, GM-CSF and IL31RA gene expression was investigated, expression could not be detected. [file Image_1.tif]

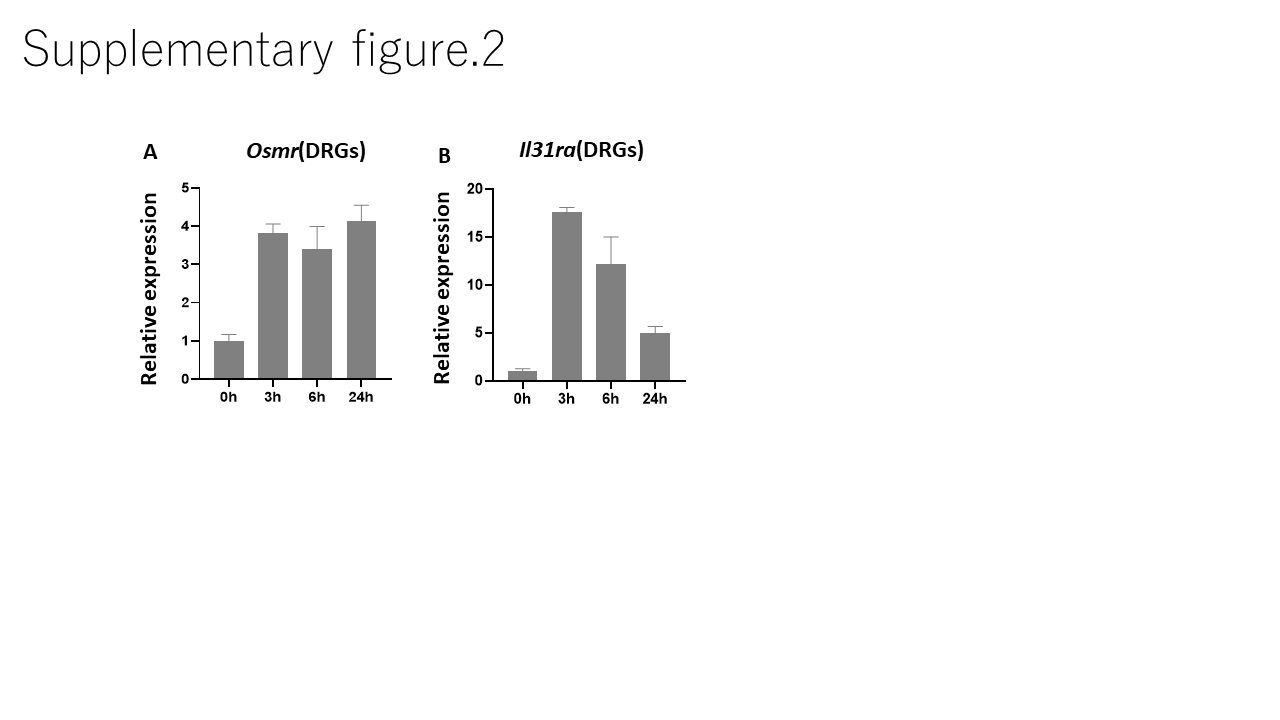

Supplement: Supplementary Figure 2 — Oncostatin M modulated the expression levels of Osmr and Il31ra in dispersed cultured mouse DRGs. (A) The expression of Osmr increased with time after OSM stimulation. (B) The expression of Il31ra increased up to 3 h and then gradually decreased below baseline. All experiments were performed ≥three times. [file Image_2.tif]
